# The association between blood selenium and metabolic syndrome in adults: a systematic review and dose–response meta-analysis of epidemiologic studies

**DOI:** 10.3389/fnut.2024.1451342

**Published:** 2025-01-15

**Authors:** Zahra Hajhashemy, Sahar Foshati, Mohammad Bagherniya, Gholamreza Askari

**Affiliations:** ^1^Student Research Committee, Isfahan University of Medical Sciences, Isfahan, Iran; ^2^Nutrition and Food Security Research Center, Department of Community Nutrition, School of Nutrition and Food Science, Isfahan University of Medical Sciences, Isfahan, Iran; ^3^Nutrition Research Center, Department of Clinical Nutrition, School of Nutrition and Food Sciences, Shiraz University of Medical Sciences, Shiraz, Iran

**Keywords:** selenium, metabolic syndrome, adults, systematic review, dose–response, meta-analysis

## Abstract

**Background and aim:**

Although the relationship between selenium and metabolic syndrome (MetS) was previously investigated, the findings were inconsistent. Therefore, we performed a systematic review and dose–response meta-analysis to summarize the association between blood selenium and MetS in adults.

**Methods:**

A comprehensive search was conducted in Medline (PubMed), ISI Web of Science, Scopus, and motor engineering of Google Scholar up to October 1st, 2024. Observational studies which reported the risk of MetS in relation to blood selenium in adults were included. The protocol of the current analysis was registered at PROSPERO as CRD42024486035.

**Results:**

Overall, 16,779 participants and 6,471 cases with MetS from 5 cross-sectional and 7 case–control studies were included in the current systematic review and meta-analysis. The findings showed that participants with the highest blood values of selenium (mean: 268.5 μg/L) in comparison to those with the lowest values (mean: 75.27 μg/L) had 40% higher risk of MetS. Nevertheless, this association was not significant (95%CI: 0.99–1.97). Due to a significant between-study heterogeneity (I^2^ = 90.4%, *p* < 0.001), subgroup analysis was conducted based on potential confounders. However, this association was only significant in a few subgroups with low number effect sizes. Linear dose–response analysis illustrated each 50 μg/L increment in circulating selenium was related to 7% higher risk of MetS (RR: 1.07, 95%CI: 0.99, 1.15) However, this association was not statistically significant. Additionally, non-linear dose–response analysis indicated a U-shaped association between blood selenium and risk of MetS with the lowest risk at 160 ug/L of blood selenium (*p* < 0.001).

**Conclusion:**

There is a U-shaped relationship between blood selenium levels risk of MetS. However, more longitudinal studies are needed to verify the causality of findings and clarify the underlying mechanisms.

## Introduction

The combination of metabolic disorders such as hypertension, hyperglycemia, hyperlipidemia, and abdominal obesity has been named metabolic syndrome (MetS) (1). MetS has a growing prevalence worldwide and patients with MetS are strongly prone to have non-communicable diseases (NCDs) such as diabetes, cardiovascular diseases (CVD), and even all-cause mortality. Environmental factors, genetics, aging, lifestyle, obesity, dietary intakes, and eating habits are leading risk factors that are involved in the MetS etiology (1–6) or incidence of its components (4, 7–12). Therefore, antioxidants and dietary intakes would have important role in controlling the metabolic health status (13–20).

Considering the role of essential metals in metabolism, evidence documented the inverse association between abnormal blood values of essential metals and NCDs (21). Selenium is one of the important essential metals that is involved in various physiological and ecological processes due to its role in the metabolism of hormones such as sexual, thyroid, and insulin (22). Additionally, selenium has a protective effect on lipid peroxidation and the structure and function of the cell membrane due to its role in glutathione peroxidase (GSH-PX), an anti-oxidant enzyme (23).

A previous meta-analysis documented that higher dietary selenium intake is protectively related to a lower risk of Mets because of its antioxidant properties (24). However, they did not investigate the association between circulating selenium and the risk of MetS. Although some previous studies illustrated a protective association between blood selenium and MetS (25), some others found a neutral or an adverse association between selenium and MetS (26–28). Furthermore, a previous systematic review investigated this subject (29); however, its findings were controversial. Moreover, due to a lack of enough eligible studies, they could not perform meta-analysis to confirm this relationship. In this regard, a systematic review and dose–response analysis were needed to exactly define the ranges of blood selenium that are protectively or adversely along with the risk of MetS. Therefore, we performed a systematic review and dose–response meta-analysis to summarize the relationship between blood selenium and MetS in adults.

## Materials and methods

We provided this study based on the Preferred Reporting Items of Systematic Reviews and Meta-Analysis (PRISMA) Guideline (30). Furthermore, the protocol of the current analysis was registered at PROSPERO^1^ as CRD42024486035.

### Search strategy

A comprehensive search was conducted in Medline (PubMed), ISI Web of Science, and Scopus, up to October 1st, 2024. Moreover, manual screening was conducted for the reference lists of eligible articles and motor engineering of Google Scholar. No limitation was considered for language or publication year. Details of mesh terms and keywords are provided in Supplementary Table 1. After including the results in Endnote software, duplicated articles were removed. Then, the title and abstract of the rest of the publications were independently screened by two investigators (ZH and SF). Any problem was dissolved through consulting by the main researcher (GA).

### Inclusion criteria

Relevant articles were included if they: (1) considered blood selenium as exposure and MetS as outcome; (2) investigated adults (≥18); and (3) reported odds ratios (ORs), relative risks (RRs), or hazard ratios (HRs) with 95% confidence intervals (CIs) for this association. Details of population, intervention/exposure, comparison/control, outcome, and study design (PICOS) criteria are provided in Table 1.

**Table 1:** PICOS criteria for inclusion of studies.

Parameter	Criteria
Participants	Adults (>18 years)
Intervention/Exposure	Higher blood Selenium
Control/Comparison	Lower blood Selenium
Outcome	Metabolic syndrome
Study design	Observational studies including prospective cohort, cross-sectional and case–control studies

### Exclusion criteria

Studies that met at least one of the following items were excluded: (1) assessed dietary selenium intakes, selenoprotein P or urine, nail, and hair selenium instead of blood selenium; (2) investigated children or adolescents; (3) reported mean ± standard deviation (SD), beta coefficient or correlation and absolute abundance instead of OR, RR, HR.

### Data extraction

The necessary information including demographic characteristics (age, gender, and target population), blood selenium, method of blood selenium or MetS assessment, related statistical tests, and their results were separately extracted by two investigators (ZH and SF). The principal investigator (GA) supervised this process.

### Quality assessment

The Newcastle-Ottawa Scale (NOS) was used to evaluate the quality of studies. Considering the domains of selection of participants, comparability, and evaluation of outcomes, a total of 10 scores were given to each cross-sectional study. Additionally, a maximum of 9 scores was assigned to each case–control study by evaluating the items of participant selection, comparability, and exposure. Details of quality assessment are provided in Supplementary Table 2.

### Statistical analysis

The overall risk of MetS in categories of selenium was estimated using the ORs/RRs/HRs and related 95%CIs. In studies that considered the last category of selenium as the reference group, the effect sizes were converted to get the risk of MetS by comparing the highest vs. lowest category of circulating selenium. To determine the between-study heterogeneity, Cochran’s Q test, I^2^, and fixed-effect models were used. Due to significant between-study heterogeneity, the overall effect was estimated by the random-effect model. If studies reported separated effect sizes based on gender, their effect sizes were merged through the fixed model to achieve an overall effect size for this study. Furthermore, the source of heterogeneity was explored through the use of subgroup analysis and meta-regression. Moreover, the individual effect of each included study was examined by performing sensitivity analysis. Additionally, Begg’s test was used to explore the publication bias. One study reported a risk of MetS per-one SD increment in blood selenium (31). Therefore, to include this study in the meta-analysis, we calculated the risk of Mets for the comparison of the third versus first tertiles of blood selenium, based on the Danesh et al. method (32). In which, the log risk estimates reported for the comparison between the highest and lowest tertiles are equivalent to 2.18 times the log risk estimates for a 1-SD increase. Furthermore, dose–response analysis was conducted based on the Greenland and Longnecker (33) and Orsini et al. (34) methods. In this method, the total number of participants, cases with MetS, the mean values of blood selenium, reported RRs/ORs/HRs, and 95% CIs of each category of circulating selenium were required. Additionally, for non-linear dose–response analysis, only studies with at least 3 categories were included. If one study did not report the median or mean values of blood selenium, the mean values of the lower and upper bounds of blood selenium were calculated. Additionally, for the highest or lowest categories with open-ended intervals, we assumed the length of adjacent intervals. A 2-stage random-effects dose–response meta-analysis was used to estimate the non-linear association between blood selenium and the risk of MetS. Nonlinear dose–response analysis was estimated through the use of the modeling of blood selenium and restricted cubic splines (three knots at fixed percentiles of 10, 50, and 90% of the distribution). Based on the Orsini et al. (34) method, the generalized least-squares trend estimation method, which takes into account the correlation within each set of reported RRs was used to calculate restricted cubic spline models. Then, all study-specific estimates were combined by using the restricted maximum likelihood method in a multivariate random-effects meta-analysis (35). The non-linear association between blood selenium and risk of MetS was estimated through the null hypothesis testing in which the coefficient of the second spline was considered equal to 0. Additionally, a 2-stage generalized least-squares trend estimation method was used to assess the linear relationship between each 50 μg/L increment in blood selenium and the risk of MetS. Such that the overall average slope was calculated through estimating the study-specific slope lines and combining them using a random-effects model (34). Analyses were conducted by STATA version 14.0. *p* values <0.05 were considered statistically significant.

## Results

### Findings from the systematic search

**Figure 1:**
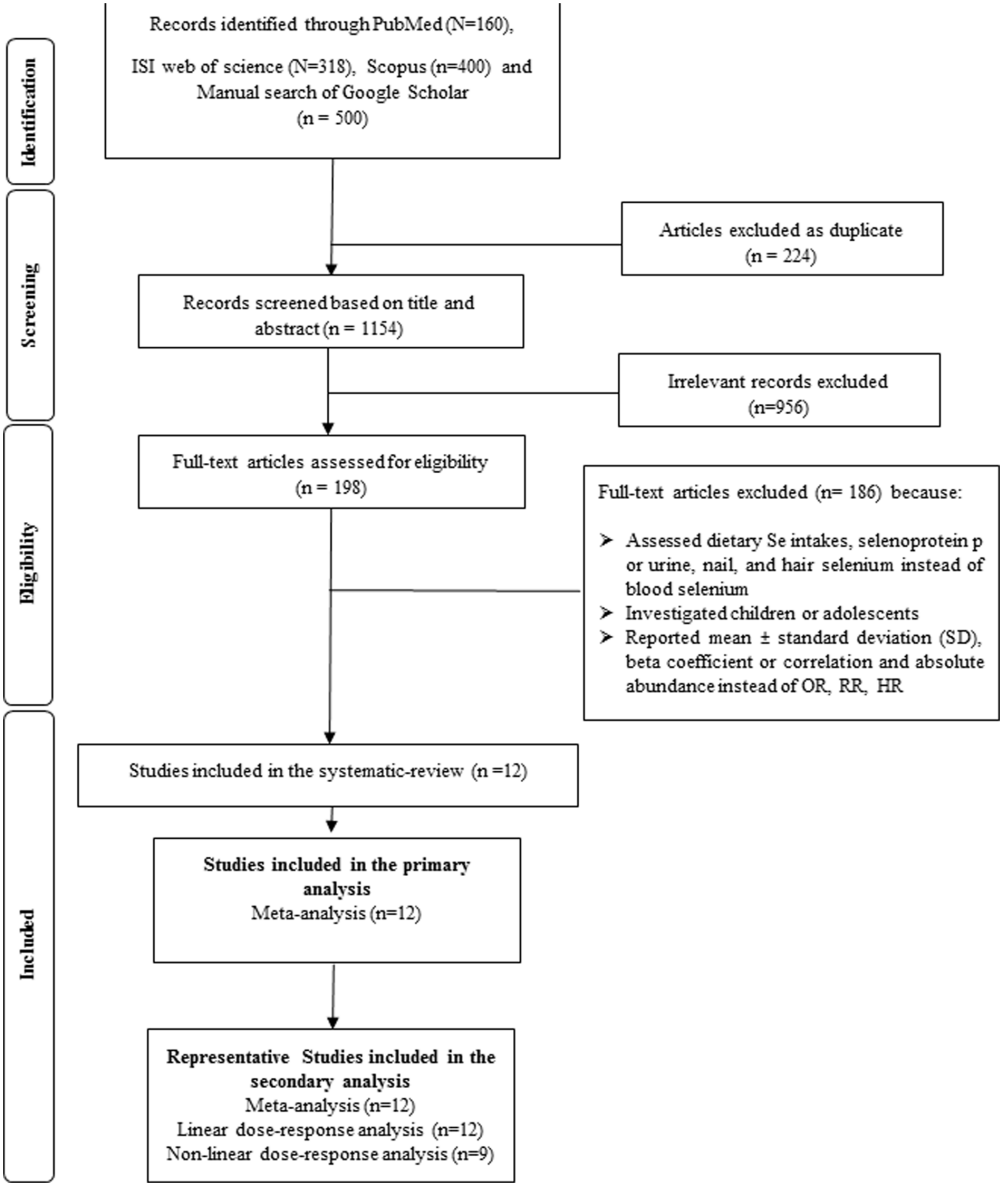
Flow diagram of search strategy and study selection.

A total of 1,378 publications resulted in the systematic search which 224 of them were duplicated. The title and abstract of 1,154 investigations were screened and after that, the full text of 198 papers were exactly assessed. Finally, 12 eligible studies were included in the systematic review. Some irrelevant studies and the reasons of excluding them are shown in Supplementary Table 3. The study selection process is provided in Figure 1.

### Study characteristics

Overall, 16,779 participants and 6,471 cases with MetS from 5 cross-sectional (31, 36–39) and 7 case–control studies (25–28, 40–42) were included in the current systematic review and meta-analysis (Table 2). These investigations were published in China (25–27, 36, 38–42), Italy (31), United States (37), and Taiwan (28) between 2012 and 2022. Nine studies (25–27, 36, 38–42) were conducted in Asian countries and the others were from non-Asian countries. Circulating selenium was assessed by inductively coupled plasma mass spectrometry (ICP-MS) (25–28, 36, 37, 39, 40, 42) and graphite furnace atomic absorption spectrometry (GFAAS) methods (26, 31, 41). Included studies defined MetS through the use of 4 methods including the Chinese diagnostic criterion for metabolic syndrome (CDS) (26, 38–41), joint interim statement (JIS) (27, 37, 42), international diabetes Federation (IDF) (25, 28, 31) and NCEP ATPШ (36). Most of the included studies had representative populations (25, 26, 28, 31, 36–39, 42). Nevertheless, 2 of them had a non-representative population (27, 41). All publications were rated as high quality because their quality scores were 7 or more. Although most of the publications adjusted their analyses for age, only 4 of them adjusted for BMI (25, 28, 37, 42).

**Table 2:** Main characteristics of included studies examined the relation between blood selenium levels and metabolic syndrome in adults.

First author, Year/(References number)	Study design/name study	Country/Latitude, °N	Age range/mean age	Sex	No. participants/cases	Selenium levels	OR/RR (95% CI)	Method (Exposure)	Definition (Outcome)	BMI	Subject	Adjustment
Pang, 2024	Cross-sectional	China	72.86	Both	852/395	Ln-transformed 1 unite increment in blood Se	1.73 (0.92,3.25)	ICP-MS	ATP Ш	NR	Elderly individuals	Gender, waistline, eGFR, age, smoke, drink, serum creatinine, health satisfaction. Lithium
						90.94 ng/mL 105.01 122.08 729.23	1 (Ref) 1.47 (0.95,2.27) 1.66 (1.07,2.57) 1.68 (1.09,2.58)					
Guo, 2023	Case–control	China	73.4	Both	191/94 203/106 190/92	≤50.2 (μg/L) 50.2–77.8 ≥77.8	1 (Ref) 1.13 (0.758, 1.68) 0.978 (0.652, 1.47)	ICP-MS	CDS	23.6	Elderly individuals	Age, sex, smoking status, drinking status.
Huang, 2022	Cross-sectional	China	59.22 ± 9.47	Both	1276/149	≤ 86.26 (μg/L) 86.26–98.75 98.75–113.32 *>* 113.32	1 (Ref) 1.43(0.86, 2.37) 0.88(0.51, 1.53) 1.21(0.72, 2.03)	ICP-MS	CDS	23.76	Mid-aged and older	Age, sex, smoking, drinking status and GFR
Zhou, 2020	Case–control (Tongji-Ezhou Cohort study)	China	55.46	Both	2558/1279	<82.36 (μg/L) 82.37–92.66 92.67–103.52 ≥103.53	0.79 (0.59–1.06) 0.75 (0.56–1.01) 0.61 (0.45–0.83) 1 (Ref)	ICP-MS	JIS	24.35	Adults	Sex, age, BMI, smoking, drinking, vigorous activity, education level
Zhang, 2020	Case–control (Beijing Population Health Cohort)	China	50–75/60	Both	4134/2095	<70.17 μg/L 70.17–81.52 81.52–93.49 >93.49	1 (Ref) 0.68(0.55–0.83) 0.52(0.42–0.63) 0.52(0.42–0.65)	ICP-MS	IDF	26.1	Adults	Education level, smoking status, alcohol intake status, BMI, PA, family history of disease,Mg, Mn, iron, Co, barium, Hg,
Bulka, 2020	Cross-sectional (NHANES 2011–2014)	USA	≥20	Both	1088/372	120.1–180.6 (μg/L) 180.7–194.9 195.0–210.0 210.1–356.0	1 (Ref) 1.20 (0.98–1.46) 1.24 (0.99–1.55) 1.31 (1.06–1.63)	ICP-MS	JIS	NR	Adults	Age, gender, race/ethnicity, and family income: poverty ratio, total caloric intake, educational attainment, smoking status, average number of drinks per day in past year, PA, survey cycle, and BMI
Feng, 2020	Cross-sectional (FAMHES) cohort	China	17–88 /37.53 ± 11.13	Men	1970/254	T1 (μg/L) T2 T3 Increase of 1 SD	1 (Ref) 0.64 (0.43–0.96) 0.77 (0.51–1.17) 1.01 (0.85–1.21)	ICP-MS	CDS	23.28	Adults	Age, smoking, alcohol drinking, PA, education status, and family history
Lu, 2019	Case-controle	Taiwan	65.8 ± 10.0	Both	292/129 290/151 292/194 291/235	≤ 76.0 μg/L 76.1–94.0 94.1–113.7 >113.7	1 (Ref) 0.82 (0.52–1.30) 1.69 (1.03–2.79) 1.66 (0.88–3.12)	ICP-MS	IDF	25.22	Outpatient department with diabetes, hypertensio, hyperlipidemia, or other chronic diseases	Age, gender,current smoking status, current drinking status, PA, BMI
Fang, 2019	Nested case–control (REACTION)	China	>40/ 63.87	Men	250/125	68.1 μg/L 79.2 90.5	1 (Ref) 0.72 (0.38–1.35) 2.72 (1.43–5.20)	GFAAS	CDS	23.92	Middle-aged and older adults	Smoking habit, alcohol consumption, PA and medication use at baseline
				Women	448/124	66.7 78.2 89.5	1 (Ref) 3.88 (2.37–6.33) 5.30 (3.31–8.74)					
Guo, 2019	Case–control	China	39 ± 12	Men	145/80	< Median (144.2 ng/mL) > 144.2	1 (Ref) 3.31 (1.4–7.82)	ICP-MS	JIS	26.4	Adults	Age
Yuan, 2015	Case–control	China	64	Both	407/204	<95.7 μg/L 95.7–176.0 ≥176	1 (Ref) 1.798 (1.064–3.039) 2.416 (1.289–4.526)	GFAAS	CDS	24.35	Adults	Age
Arnaud, 2012	Cross-sectional (The IMMIDIET study)	Italy, Belgium and England	45.62	Men Women	942/194 960/99	Increase of 1 SD (Men: 0.23 mmoL/L,Women: 0.24 mmoL/L)	0.97 (0.81–1.16) 1.33 (1.06–1.67)	GFAAS	IDF	NR		Age, country group, social status, PA, energy intake, alcohol consumption and smoking and in women additionally adjusted for menopausal status, uses of oral contraceptive pills or hormonal replacement therapy

Ref, Reference; ORs, odds ratios; RR, relative risk; CIs, confidence intervals; ICP-MS, inductively coupled plasma mass spectrometry; GFAAS, graphite furnace atomic absorption spectrometry; CDS, Chinese diagnostic criterion for metabolic syndrome; JIS, joint interim statement; IDF, International Diabetes Federation; GFR, Glomerular filtration rate; BMI, body mass index; PA, physical activity; Mg, Magnesium; Mn, Manganese; NHANES, National health and nutrition examination surveys; FAMHES, Fangchenggang Area Male Health and Examination Survey; NR, not reported.

### Finding from meta-analysis of highest versus lowest level of blood selenium in relation to MetS

**Figure 2:**
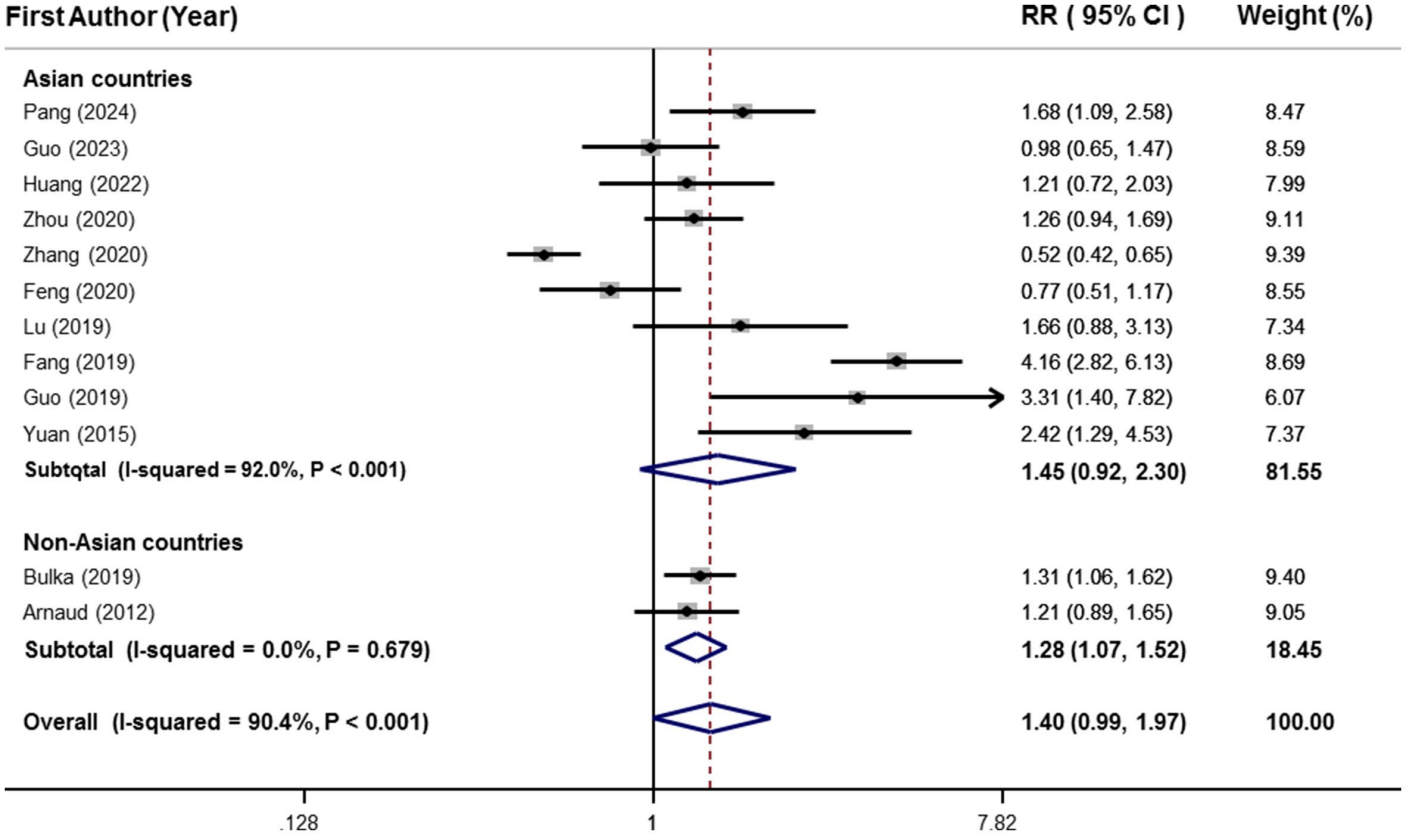
Forest plots of the relationship between blood selenium levels and metabolic syndrome in adults, stratified by study location.

Twelve effect sizes (including 16,779 participants and 6,471 cases with MetS from 12 publications) (25–28, 31, 36–42) investigated the relationship between circulating selenium and MetS in adults and were included in the meta-analysis. The findings of meta-analysis showed that participants with the highest blood values of selenium (mean: 268.5 μg/L) in comparison to those with the lowest values (mean: 75.27 μg/L) had 40% higher risk of MetS. Nevertheless, this association was not statistically significant (95%CI: 0.99–1.97), as shown in Figure 2.

Due to a significant between-study heterogeneity (I^2^ = 90.4%, *p* < 0.001), subgroup analysis was conducted based on study location (Figure 2). Although highest vs. lowest blood selenium was significantly related to 28% higher risk of MetS (95%CI: 1.07–1.52) in the subgroup of non-Asian countries, there was not any significant association in the subgroup of Asian countries (RR:1.45; 95%CI: 0.92–2.30). Moreover, heterogeneity was completely removed in the subgroup of non-Asian countries (I^2^ = 0.0%, *p* = 0.67); however, it was kept significant in the subgroup of Asian countries (I^2^ = 92.0%, *p* < 0.001), as shown in Figure 2. Details of subgroup analysis based on other confounders are provided in Table 3. There was a significant and straight association between blood selenium and risk of MetS in the subgroups with low number of effect sizes including non-Asian countries, developed countries, NCEP-ATP Ш, JIS, and sufficient vs. deficient.

**Table 3:** Results of subgroup-analysis for circulating selenium levels and risk of MetS in adults.

	No. of effect sizes	RR (95% CI)	P within^1^	I^2^ (%)	P between^2^
Overall	12	1.40 (0.99, 1.97)	<0.001	90.4	
Study design					0.16
Cross-sectional studies	5	1.21 (0.98, 1.50)	0.12	44.8	
Case–control studies	7	1.62 (0.85, 3.05)	<0.001	94.3	
Methods of circulating selenium assessment					<0.001
ICP-MS	9	1.17 (0.84, 1.64)	<0.001	86.8	
GFAAS	3	2.28 (0.98, 5.31)	<0.001	91.8	
Development status of countries					0.066
Developed	3	1.30 (1.1, 1.54)	0.67	0.0	
Developing	9	1.43 (0.88, 2.34)	<0.001	92.8	
Study location					0.13
Asian countries	8	1.45 (0.92, 2.30)	<0.001	92.2	
Non-Asian countries	2	1.28 (1.07, 1.52)	0.67	0.0	
Representativeness of study population					0.11
Representative	9	1.29 (0.87, 1.93)	<0.001	92.2	
Non-Representative	2	1.87 (0.86, 4.07)	0.008	79.3	
MetS definition					<0.001
NCEP-ATP Ш	1	1.68 (1.09, 2.58)	–	–	
CDS	5	1.55 (0.79, 3.04)	<0.001	90.8	
JIS	3	1.42 (1.05, 1.93)	0.10	55.1	
IDF	3	0.98 (0.48, 2.0)	<0.001	92.3	
Selenium categories					<0.001
T3 vs. T1	5	1.54 (0.84, 2.85)	<0.001	91.1	
Q4 vs. Q1	6	1.16 (0.76, 1.79)	<0.001	90.3	
Sufficient vs. Deficient	1	3.31 (1.40, 7.82)	–	–	
Gender					0.51
Both	10	1.40 (0.96, 2.04)	<0.001	91.4	
Male	2	1.52 (0.36, 6.31)	0.003	88.8	

1 P for heterogeneity, within subgroup.

2 P for heterogeneity, between subgroups.

MetS, metabolic syndrome; BMI, body mass index; ICP-MS, inductively coupled plasma mass spectrometry; GFAAS, graphite furnace atomic absorption spectrometry; CDS, Chinese diagnostic criterion for metabolic syndrome; JIS, joint interim statement; IDF, International Diabetes Federation.

Moreover, meta-regression was conducted based on the mean age of participants (*β* = 0.0057, *p* = 0.71, I^2^ residual = 91.25%) and quality score of eligible investigations (*β* = −0.264, *p* = 0.15, I^2^ residual = 91.21%). However, none of them had a significant effect on the overall estimate. Furthermore, based on sensitivity analysis, the overall effect was not influenced by any included studies. Additionally, according to Begg’s plot and Begg’s test (*p* = 0.27), there was no evidence of publication bias.

### Findings from dose–response analysis

**Figure 3:**
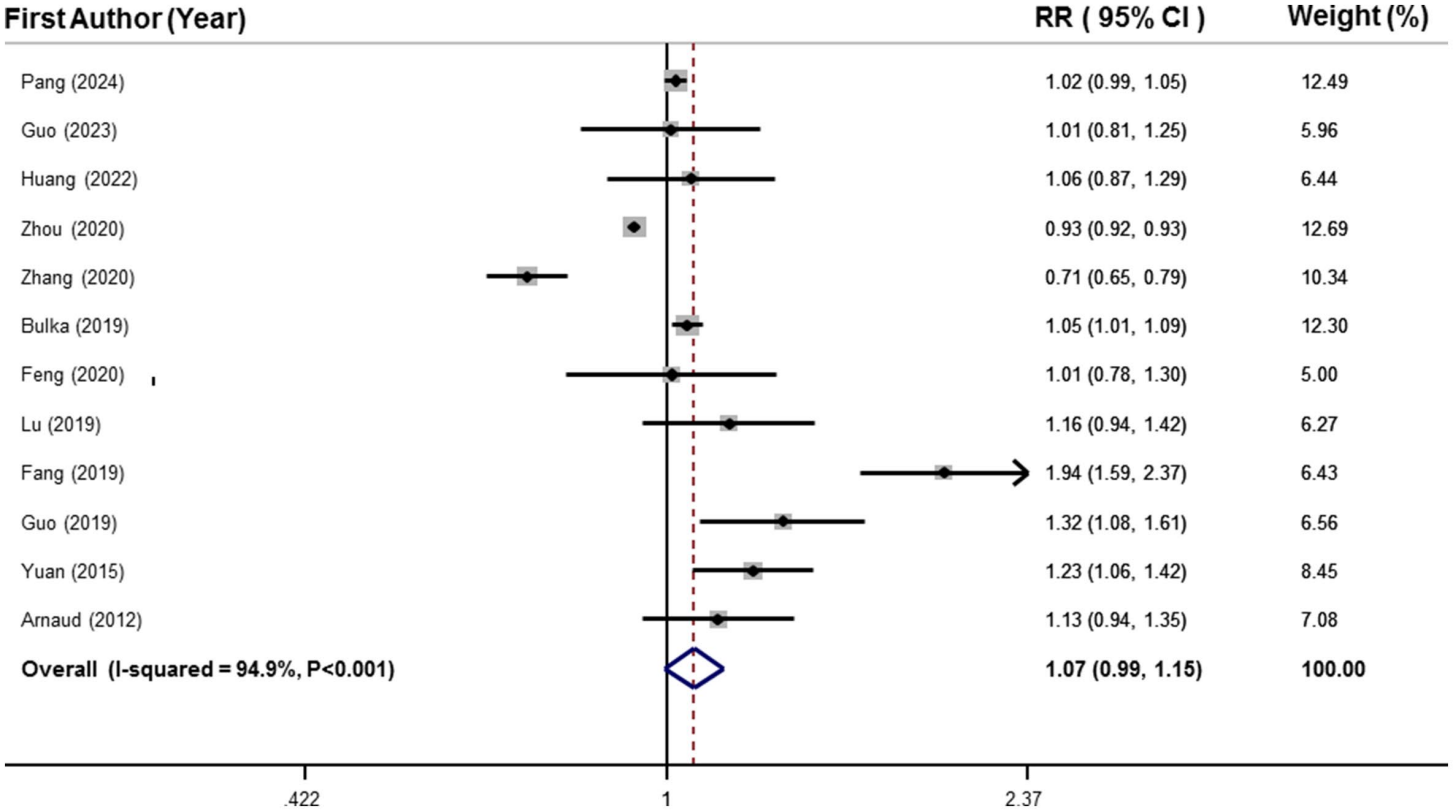
Forest plots of the linear relationship between blood selenium levels and metabolic syndrome.

Data from 16,779 participants and 6,471 cases with MetS from 12 studies were included in the linear dose–response analysis (25–28, 31, 36–42). Results showed each 50 μg/L increment in circulating selenium was related to 7% higher risk of MetS (RR: 1.07, 95%CI: 0.99, 1.15), as shown in Figure 3. However, this association was not statistically significant.

**Figure 4:**
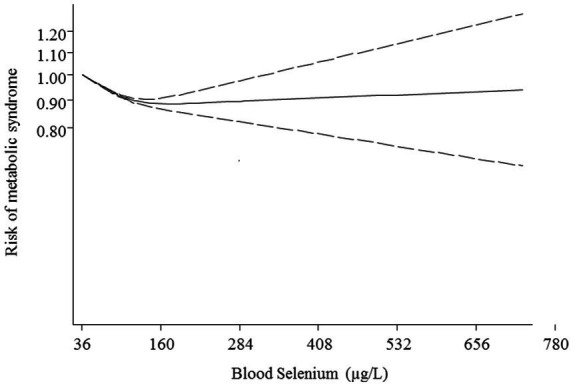
The non-linear relationship between blood selenium levels and metabolic syndrome.

Additionally, non-linear dose–response analysis was conducted on 9 studies including 12,762 participants and 5,844 cases with MetS (25, 26, 28, 36, 37, 39–42). Findings indicated a U-shaped association between blood selenium and risk of MetS with the lowest risk at 160 μg/L of blood selenium (*p* < 0.001; Figure 4).

## Discussion

The results of this systematic review and dose–response meta-analysis of observational studies revealed that although the highest versus lowest concentration of blood selenium was related to higher risk of MetS, this association was not statistically significant. After performing subgroup analysis, this relationship was significant only in the subgroups with low number of effect sizes. Additionally, the findings of the linear dose–response analysis indicated a direct association between each 50 μg/L increment in blood selenium and the risk of MetS. However, this relationship was not statistically significant. Furthermore, non-linear dose–response analysis illustrated a U-shaped association between blood selenium values and risk of MetS, with the lowest risk at 160 μg/L of blood selenium.

To the best of our knowledge, the current meta-analysis is the first one that investigated the association between blood selenium levels and the risk of MetS. Interestingly, the findings obtained from multiple studies support the protective role of selenium in the etiology of MetS. For instance, prior to this study, two meta-analyses of three case–control studies found that patients with MetS had significantly lower serum levels of selenoprotein P than controls (43, 44). In addition, another meta-analysis of four cross-sectional studies reported a significant inverse relationship between dietary selenium intake and the risk of MetS (24). While interpreting the findings of these three meta-analyses, we should consider the nature of the exposures used in the aforesaid meta-analyses. Selenoprotein P is a negative acute-phase reactant that its serum levels can be reduced in inflammatory states such as MetS (43, 45). Besides, selenoprotein P is produced by hepatocyte. Moreover, patients with MetS have been indicated to suffer from impaired liver function; therefore, a reduction in selenoprotein synthetic capacity is probable in MetS (43, 46, 47). Moreover, dietary selenium intake, estimated from a food frequency questionnaire or a 3-day or 24-h recall (24), is subject to a high degree of bias and cannot appropriately predict selenium status (48). In contrast to selenoprotein P and also dietary selenium intake, selenium concentration in blood (plasma, serum, or whole blood) is a reliable indicator of selenium status (48, 49).

On the other hand, the findings of some meta-analyses confirmed the adverse relationship between selenium and the risk of diseases. For instance, recent meta-analyses showed a significantly positive and roughly linear relationship between blood selenium levels and the risk of diabetes mellitus (50–52). It is worth mentioning that the prevalence of MetS is very high in the diabetic population, particularly among female patients (53). Furthermore, observational surveys indicated significant positive associations between blood selenium concentrations and the risk of MetS components including insulin resistance (54), hyperglycemia (55), dyslipidemia (55, 56), hypertension (57, 58), and central obesity (59). In addition, some meta-analyses of randomized controlled trials revealed that selenium supplementation significantly increased systolic blood pressure (60), tumor necrosis factor-alpha (an inflammatory trigger for insulin resistance) (61), and low-density lipoprotein cholesterol (60). Additionally, a study from National Health and Nutrition Examination Survey (NHANES) Ш indicated a U-shaped relationship between serum selenium and cardiovascular mortality (62). In the current study, we also revealed a U-shaped association between circulation selenium and risk of MetS with the lowest risk at 160 μg/L of blood selenium. Therefore, it seems that the relationship between blood selenium and NCDs is dose-dependent. Although the normal range of blood selenium (120–160 μg/L) is protectively related to a lower risk of MetS, the toxic values of circulating selenium might increase the risk of MetS.

The protective relationship between blood selenium and MetS may be due to the antioxidant properties of selenium. Inflammation and oxidative stress are the leading causes of MetS and its components (63). On the other hand, selenium is one of the components of glutathione peroxidase (GSH-PX) and the process of antioxidant defense. Therefore, blood values of selenium are protectively related to a lower risk of MetS due to its antioxidant features (64). However, potential biochemical mechanisms underlying the direct relationship between toxic blood selenium levels and MetS risk have not been well elucidated. As a known fact, the range of selenium status is very narrow from deficiency to sufficiency to toxicity (65). Therefore, it seems that blood selenium can easily reach excessive levels and cause detrimental effects on human health (66). In detail, excess blood levels of selenium (especially inorganic selenium) may lead to oxidative stress by increasing the production of various reactive oxygen and nitrogen species (42). It is worth noting that oxidative stress is a central pathophysiological feature of MetS (63). In addition, high blood levels of selenium can affect the expression of protein tyrosine phosphatase and result in insulin resistance, diabetes mellitus, and obesity (67, 68). Moreover, it has been shown that high blood selenium concentrations may increase protein biosynthesis, gluconeogenesis, cholesterol biosynthesis, and lipogenesis and decrease glycolysis and cholesterol hydrolysis in the liver (69). Furthermore, high blood selenium can deplete chromium (70), and chromium deficiency may elevate blood pressure by increasing renin-angiotensin system activity and reducing nitric oxide system activity (65, 71).

The results of subgroup analyses implied that there was geographical difference in the association between blood selenium concentrations and the risk of MetS. The geographical difference may be due to variations in the selenium content of soil, water, plants, and air across different countries. As an example, people living in industrially developed countries have higher inhalation exposure to selenium than others (72). Additionally, due to different lifestyles in countries, the prevalence of MetS and its components varies in different regions (73).

### Strengths and limitations

This study has multiple strengths. First, to the best of our knowledge, this is the first meta-analysis investigating the relationship between blood selenium levels and the risk of MetS. Second, several subgroup analyses and meta-regressions were conducted to explore potential sources of heterogeneity between the included studies. Third, a dose–response analysis was performed to clarify the quantitative estimation of the above association.

However, there are several limitations that warrant attention. The number of included studies was relatively small. Hence, more investigations especially with prospective design are needed to confirm the causality of this association. Moreover, there was substantial heterogeneity in this meta-analysis that could not be fully resolved by subgroup analyses and meta-regressions. This may be due to within and between-subject biological variations in blood selenium (75), the presence of multiple single nucleotide polymorphisms in selenium metabolism-related genes (66), the multi-causality and complex genetic and phenotypic nature of MetS (76), and the different selenium content of foods, soul and even air and water in different countries (72). Furthermore, the design of all included studies was cross-sectional or case–control; therefore, a cause-and-effect relationship could not be established. Additionally, most of the included studies originated from Eastern countries, therefore the findings could not be generalizable to Western populations.

### Implications for practice and research

The results of this meta-analysis suggested that there is a U-shaped relationship between circulating selenium and the risk of MetS. Although the increasing blood selenium from 36 up to 160 μg/L was along with a reduction risk of MetS, the values more than 160 μg/L were related to higher risk of MetS. Therefore, it is important to intake enough selenium through the diet; however, it is necessary to avoid selenium supplementation in people without confirmed deficiency. Moreover, conducting prospective cohort studies is required to confirm our findings and establish causation.

## Conclusion

In a nutshell, there is a U-shaped relationship between blood selenium levels risk of adult MetS. However, more longitudinal studies are needed to verify the causality of findings and clarify the underlying mechanisms.

## Data Availability

All the included data in the analysis are provided in the paper (Table 2). Further inquiries can be directed to the corresponding author.

## Footnotes

^1^ http://www.crd.york.ac.uk/PROSPERO
