# Supplementary material for: The association between blood selenium and metabolic syndrome in adults: a systematic review and dose–response meta-analysis of epidemiologic studies
Source: Front Nutr. 2025 Jan 15;11:1451342. doi: 10.3389/fnut.2024.1451342 (PMC11775477; doi:10.3389/fnut.2024.1451342)
Supplement: Supplementary file 1 [file Data_Sheet_1.pdf]

**Supplemental Table 1**. MeSH and non-MeSH terms that were used in the systematic search		
Pubmed	(("Syndrome X" OR "metabolic syndrome" OR " metabolic syndromes" OR " insulin resistance syndrome")) AND (("selenium" OR "selenite" OR "selenate" OR "seleno"))	160
Scopus	(TITLE-ABS-KEY (("Syndrome X" OR "metabolic syndrome" OR " metabolic syndromes" OR " insulin resistance syndrome")) AND TITLE-ABS-KEY (("selenium" OR "selenite" OR "selenate" OR "seleno")))	400
WOS	("Syndrome X" OR "metabolic syndrome" OR " metabolic syndromes" OR " insulin resistance syndrome") (Topic) AND (“selenium” OR “selenite” OR “selenate” OR “seleno”) (Topic)	318

Supplemental Table 2. Details of quality assessment of included studies in the systematic review and met-analysis based on Newcastle-Ottawa Scale[1].

A. Cross-sectional studies

	Representativeness of the sample	Sample size	Non-respondents	Ascertainment of the exposure (risk factor)	Comparability of subjects in different outcome groups	Assessment of outcome	Statistical test	Total score
Pang, 2024	*	*	*	**	**	**	*	10
Huang et al, 2022	*	*	*	**	**	**	*	10
Bulka et al, 2020	*	*	*	**	**	**	*	10
Feng et al, 2020	*	*	*	**	**	**	*	10
Arnaud et al, 2012	*	*	*	**	**	**	*	10

B. Case-control studies

	Is the case definition adequate	Representativeness of the cases	Selection of Controls	Definition of Controls	Comparability of cases and controls on the basis of the design or analysis	Ascertainment of exposure	Same method of ascertainment for cases and controls	Non-Response rate	Total score
Guo et al, 2023	*	*	*	*	**	*	*	*	9
Zhou et al, 2020	*	*	*	*	**	*	*	*	9
Zhang et al, 2020	*	*	*	*	**	*	*	*	9
Fang et al, 2019	*	*	*	*	**	*	*	*	9
Guo et al, 2019	*		*	*	*	*	*	*	7
Lu et al, 2019	*	*	*	*	**	*	*	*	9
Yuan et al, 2015	*		*	*	*	*	*	*	7

[1]Wells GA, Shea B, O'Connell D, Peterson J, Welch V, Tugwell P. The Newcastle-Ottawa Scale (NOS) for Assessing the Quality of Nonrandomised Studies in Meta-Analyses. Available from: http://www.ohri.ca/programs/clinical_epidemiology/oxford.asp

Supplemental Table 3. Details of more relevant studies that were excluded.

	First author (Year)	Title of article	Reason of exclusion from current meta-analysis
(1)	Chen, 2021	The Relationship between Metabolic Syndrome and Plasma Metals Modified by EGFR and TNF-_ Gene Polymorphisms	Did not report 95%CI
(2)	Gharipour, 2017	Association of Expression of Selenoprotein P in mRNA and Protein Levels with Metabolic Syndrome in Subjects with Cardiovascular Disease: Results of Selenegene Study	Reported relationship between Selenoprotein P and metabolic syndrome
(3)	Jang, 2018	Correlates of toenail selenium and its cross-sectional association with metabolic syndrome	Reported relationship between toenail selenium and metabolic syndrome
(4)	Ma, 2020	Associations between essential metals exposure and metabolic syndrome (MetS): Exploring the mediating role of systemic inflammation in a general Chinese population	Reported relationship between urinary selenium and metabolic syndrome
(5)	Ford, 2003	The Metabolic Syndrome and Antioxidant Concentrations	Reported regression coefficient for the relationship between blood selenium and metabolic syndrome
(6)	Li, 2023	A causal relationship between antioxidants, minerals and vitamins and metabolic syndrome traits: a Mendelian randomization study	Its design was Mendelian randomization study

REFERENCES

1. Chen T-H, Kung W-S, Sun H-Y, Huang J-J, Lu J-Y, Luo K-H, et al. The relationship between metabolic syndrome and plasma metals modified by EGFR and TNF-α gene polymorphisms. Toxics. 2021;9(9):225.
2. Gharipour M, Sadeghi M, Salehi M, Behmanesh M, Khosravi E, Dianatkhah M, et al. Association of expression of selenoprotein P in mRNA and protein levels with metabolic syndrome in subjects with cardiovascular disease: Results of the Selenegene study. The Journal of Gene Medicine. 2017;19(3):e2945.
3. Jang H, Morris J, Park K. Correlates of toenail selenium and its cross-sectional association with metabolic syndrome. J Hum Nutr Diet. 2018;31(5):603-11.
4. Ma J, Zhou Y, Wang D, Guo Y, Wang B, Xu Y, et al. Associations between essential metals exposure and metabolic syndrome (MetS): Exploring the mediating role of systemic inflammation in a general Chinese population. Environ Int. 2020;140:105802.
5. Ford ES, Mokdad AH, Giles WH, Brown DW. The metabolic syndrome and antioxidant concentrations: findings from the Third National Health and Nutrition Examination Survey. Diabetes. 2003;52(9):2346-52.
6. Li J, Song F. A causal relationship between antioxidants, minerals and vitamins and metabolic syndrome traits: a Mendelian randomization study. Diabetology & Metabolic Syndrome. 2023;15(1):194.
